# Process Development for the Detoxification of Fermentation Inhibitors from Acid Pretreated Microalgae Hydrolysate

**DOI:** 10.3390/molecules26092435

**Published:** 2021-04-22

**Authors:** Ji-Woo Hong, Da-Hye Gam, Jun-Hee Kim, Sung-Jin Jeon, Ho-Seob Kim, Jin-Woo Kim

**Affiliations:** 1 Department of Food Science, Natural Science Building #118, Sun Moon University, 70 Sunmoon-ro 221, Tangjeong-myeon, Asan-si, Chungnam 336-708, Korea; hgw130@naver.com (J.-W.H.); ank7895@naver.com (D.-H.G.); jun981014@naver.com (J.-H.K.); zzx01010@naver.com (S.-J.J.); 2 Department of Physics and Nano-Science, Natural Science Building #415, Sun Moon University, 70 Sunmoon-ro 221, Tangjeong-myeon, Asan-si, Chungnam 336-708, Korea; hskim3@sunmoon.ac.kr; 3 CEBT Co. Ltd., Sun Moon University, 70 Sunmoon-ro 221, Tangjeong-myeon, Asan-si, Chungnam 336-708, Korea; 4 FlexPro Biotech. Co. Ltd., Business Start-Up Center #309, 70 Sunmoon-ro 221, Tangjeong-Myeon, Asan-si, Chungnam 336-708, Korea

**Keywords:** microalgae, pretreatment, hydrolysate, 5-hydroxymethyl furfural, furfural, detoxification, fermentation

## Abstract

The aim of this study was to remove 5-hydroxymethyl furfural (5-HMF) and furfural, known as fermentation inhibitors, in acid pretreated hydrolysates (APH) obtained from *Scenedesmus obliquus* using activated carbon. Microwave-assisted pretreatment was used to produce APH containing glucose, xylose, and fermentation inhibitors (5-HMF, furfural). The response surface methodology was applied to optimize key detoxification variables such as temperature (16.5–58.5 °C), time (0.5–5.5 h), and solid–liquid (S-L) ratio of activated carbon (0.6–7.4 *w/v*%). Three variables showed significant effects on the removal of fermentation inhibitors. The optimum detoxification conditions with the maximum removal of fermentation inhibitors and the minimum loss of sugars (glucose and xylose) were as follows: temperature of 36.6 °C, extraction time of 3.86 h, and S-L ratio of 3.3 *w/v*%. Under these conditions, removal of 5-HMF, furfural, and sugars were 71.6, 83.1, and 2.44%, respectively, which agreed closely with the predicted values. When the APH and detoxified APH were used for ethanol fermentation by *S. cerevisiae*, the ethanol produced was 38.5% and 84.5% of the theoretical yields, respectively, which confirmed that detoxification using activated carbon was effective in removing fermentation inhibitors and increasing fermentation yield without significant removal of fermentable sugars.

## 1. Introduction

With the rapid industrialization and population growth over the past few decades, consumption of fossil fuels has dramatically increased, and pollution and energy depletion have become a threat to the environment as well as to humans. To solve this problem, renewable energy is being actively developed to replace fossil fuels. Among various types of renewable energy, bioethanol has great potential to reduce greenhouse gas emissions and dependence on fossil fuels [1]. Bioethanol is advantageous over other renewable energies in that it is readily available through mixing directly with conventional fossil fuels and is known as the only alternative energy source available in the transportation sector without major changes to fossil fuel-based combustion engines and energy supply infrastructure [2,3]. In the past, much attention has been paid to the production of bioethanol using wood-based biomass, but recent bioethanol research has focused on the use of microalgae called third-generation biomass.

Microalgae have high biological diversity, with approximately 25,000 species, and have been receiving much attention because of their rapid growth and environmental advantages; they can be mass produced, are free from ethical problems caused by conflicts with food resources, grow fast, and have excellent carbon fixation ability [4,5]. Many microalgae strains can accumulate 20–80% of lipids, while others can accumulate up to 70% as carbohydrates, which are insoluble polysaccharides such as cellulose, hemicellulose, pectin, and starch [6]. In the production of second-generation biofuels from lignocellulosic feedstock, two polysaccharides, cellulose and hemicellulose, need to be converted each into glucose and xylose through pretreatment and enzyme hydrolysis [7]. Due to the complex structure and robustness of lignocellulose caused mainly by lignin and cellulose, pretreatment is an essential step for the production of fermentable sugar through cellulose hydrolysis by removing lignin, reducing the cellulose crystal structure, and increasing the accessibility of enzyme to cellulose for the production of fermentable sugar [8]. The high content of carbohydrates makes microalgae an attractive feedstock for bioethanol production. The cell walls of microalgae are characterized by low lignin content and are mainly composed of cellulose. Additionally, starch is a carbohydrate produced by photosynthesis and is present in large amounts in microalgae [9]. The pretreatment and enzymatic hydrolysis processes of microalgae can be minimized compared to lignocellulosic feedstock due to its low lignin content. Therefore, carbohydrates produced from microalgae can be easily saccharified; microalgae are recognized as a highly competitive feedstock for biofuel production because they require simplified pretreatment [10,11].

By the hydrolysis of lignocellulosic feedstock through chemical pretreatment, components of microalgae’s cell wall, cellulose and hemicellulose, are hydrolyzed into monomeric sugars such as glucose and xylose that can be fermented by microorganisms [12]. Traditional chemical pretreatments of lignocellulosic feedstock include alkali, acid, organic solvent, and ionic liquid, among which dilute acid is most widely used due to low process cost, high efficiency, and simplicity. However, since dilute acid pretreatment takes place in acidic conditions under high temperature, the glucose and xylose produced are non-selectively converted into fermentation inhibitors such as 5-hydroxymethyl furfural (5-HMF) and furfural [13]. These inhibitors have been reported to significantly reduce ethanol fermentation yield by reducing enzyme activity and microbial growth. Thus, a preliminary process for removing fermentation inhibitors called detoxification is essential for the production of useful substances using acid pretreated hydrolysate (APH) through fermentation [14]. Among the various detoxification processes including distillation, organic solvent extraction, lime precipitation, ion-exchange chromatography, and activated carbon absorption, the removal of toxic byproducts using activated carbon is known to be more economical and easy to apply to industries [15,16,17].

The aim of this study was to optimize the detoxification process conditions using response surface methodology (RSM), a statistically based optimization, to effectively remove 5-HMF and furfural by applying activated carbon and increase the yield of ethanol fermentation. To prove the detoxification effect of activated carbon, we optimized the key detoxification process variables including temperature, time, and solid–liquid (S-L) ratios, and evaluate the effect of increasing the fermentation yield on the produced detoxified APH.

## 2. Results and Discussion

### 2.1. Composition Analysis

When the composition of *S. obliquus* was analyzed, the most abundant components were 30.8% cellulose and 13.8% starch, consisting of β-1,4 and α-1,4-linked glucose, respectively (Table 1). By contrast, hemicellulose, the second most abundant carbohydrate on Earth, was measured at only 4.9%, which was lower compared to both cellulose and starch. In terrestrial plants, lignocellulose is generally composed of cellulose (30–60%), hemicellulose (15–40%), and lignin (5–25%), whereas the content of cellulose and hemicellulose in *S. obliquus* was lower than that in terrestrial plants, making it unfavorable as feedstock for bioethanol production due to the lower production of fermentable sugar. However, *S. obliquus* had 13.8% starch, which was significantly higher than that in terrestrial plants, providing an advantage in the production of fermentable sugars. The content of lignin, known to inhibit fermentation and enzymatic hydrolysis by producing phenolics during pretreatment, was at 1.9%; thus, pretreatments for removing lignin and degrading rigid cell walls could be simplified, resulting in energy and process cost savings (Table 2). Total sugar yields after microwave-assisted pretreatment (MAP) of *S. obliquus* are shown in Table 1. Through MAP, a total of 77.8% of the cellulose and starch was hydrolyzed to glucose and 61.7% of the hemicellulose was converted to xylose. The generation of furfural was very high compared to 5-HMF and led to a lower conversion rate of xylose (61.7%) compared with glucose (77.8%), which was attributed to the rapid degradation of xylose to furfural through an acid catalyzed pretreatment at high temperature. 

### 2.2. Model Fitting

To obtain the maximum value of the target response, it is essential to conduct the optimization experiments on the main process variables. Response surface methodology (RSM) is an optimization method that analyzes the complex relationship between independent and response variables. One of the RSMs, central composite design (CCD), is the most widely used statistical method based on the multivariate nonlinear model for the optimization of process variables, and was applied to determine the regression model equations for the prediction. For multivariate optimization, CCD offers the advantage of enabling effective and time-saving optimization by reducing the total number of experimental runs required over the conventional one-factor-at-a-time method [19].

When detoxifying fermentation inhibitors in APH using activated carbon, a decrease in fermentation yield occurs due to the non-specific adsorption of fermentable sugars. In order to increase the utilization of AHP by increasing fermentation yield, it is necessary to develop a process that maximizes the removal of fermentation inhibitors and minimizes sugar loss at the same time. Fitting the model is essential for interpreting the accuracy of CCD for predicting optimal conditions and responses. We selected three variables for detoxification, namely extraction temperature (X_1_), extraction time (X_2_), and S-L ratio (X_3_), as independent variables and 5-HMF removal, furfural removal, and sugar loss as responses. The lower and upper limits of the detoxification variable were determined according to our previous experiments as shown in Table 3 [20,21].

Based on CCD results obtained from the experimental data, quadratic models were developed to reveal the correlation between independent and dependent variables (Table 4). 

Where Y is the yield of responses and X_1_, X_2_, and X_3_ are the coded variables for independent variables. X_1_: extraction temperature (°C); X_2_: extraction time (h); X_3_: S-L ratio (%); Y_1_: 5-HMF removal (%); Y_2_: furfural removal (%); Y_3_: sugar loss (%); R^2^ = coefficient of determination; P = probability value. 

The adequacy of the models was justified using analysis of variance (ANOVA) and values for the removal rates of 5-HMF, furfural, and sugar are listed in Table 4. The model F-value is the ratio of mean squared for the individual term to the mean squared for the residual, and the variables having F-statistics probability value less than 0.05 are considered to be significant [22]. Table 5 demonstrates that the models were highly significant, as evident from the low probability of *p* > F values in regressions. 

### 2.3. Perturbation Curves

**Figure 1:**
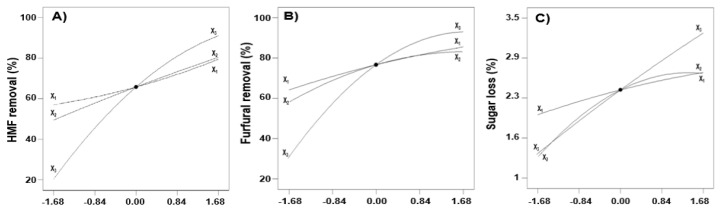
Effect of independent variables on 5-HMF removal (**A**), furfural removal (**B**), and sugar loss (**C**). Each level of variable was expressed as code values. Extraction temperature (X_1_); extraction time (X_2_); S-L ratio (X_3_).

Using a second-order regression model, optimal conditions for detoxification can be found by exploring the design space and visualizing the relationship between variables and responses. The perturbation curve exhibits how a function of a dependent variable responded as the level of independent variable changed when other independent variables were fixed at their center point; a steep slope or curvature in the plot indicates the sensitivity of the dependent response variable [23]. The removal of 5-HMF and furfural was plotted by changing only one variable over its range while other variables were held constant (Figure 1A,B, respectively). For 5-HMF and furfural removal, an increase in removal rates was observed as temperature, time, and S-L ratio were increased. This confirmed that all independent variables had significant effects on the removal of fermentation inhibitors, as shown by the results of the ANOVA. In perturbation curves, effects of the independent variables during the removal of fermentation inhibitors were in the order of S-L ratio > time > temperature. The steep curvature of the S-L ratio indicated that the removal of fermentation inhibitors was highly affected by the amount of activated carbon added in APH. The effect of each variable on sugar loss is shown in Figure 1C. Sugar losses increased when each variable increased, and about 2.5% of the sugar was removed during the detoxification process, which was very low compared to 70–90% of the fermentation inhibitor removal rate. This trend was similar to that observed for the removal of fermentation inhibitors, confirming that sugar and fermentation inhibitors were not selectively adsorbed by activated carbon.

### 2.4. Maximizing 5-HMF and Furfural Removal 

5-HMF and furfural, organic compounds containing furan rings, are produced when glucose and xylose are pretreated at high temperatures under acidic condition and reduce ethanol yield by inhibiting microorganism growth during fermentation [24,25]. Therefore, detoxification process is important and the process conditions were optimized by applying 3-variable and 5-lelvel CCD with 17 runs (Table 6) to maximize the detoxification of HMF and furfural (Table 6).

When experimental values for each condition were measured, 5-HMF and furfural removal rates ranged from 20.1% to 97.4% and 28.3% to 96.1%, respectively. The maximum removal rate of fermentation inhibitors was achieved at 50 °C, 4.5 h, and 6.0% (run no. 8), with highest removal rates of 97.4% of HMF and 96.1% of furfural. As derived by the Design-Expert ^®^ software 8.0 (State-Ease, Minneapolis, MN, USA), second-order regression equations were developed and predicted the responses for 5-HMF removal, furfural removal, and sugar loss (Table 4). In second-order regression equations, the positive coefficient of the primary term represents the proportional relationship between the variable and the response. When the effect of three independent variables in regression equations was evaluated, removal rates of 5-HMF and furfural increased proportional to the level of independent variables. Statistical analysis of results was conducted using ANOVA (Table 5). Model F-values of 27.76, 22.35, and 5.47 and *p*-values of 0.0001, 0.0002, and 0.0178, respectively, indicated that the models were significant, and that there was a < 1.78% chance that a “Model F-value” this large could occur due to noise. In this study X_1_, X_2_, and X_3_ were all determined as significant terms. Among three independent variables, the removal of fermentation inhibitors increased the most as the S-L ratio increased (*p* < 0.0001). R^2^ is defined as the ratio of the sum of squares due to regression to the total sum of squares and is interpreted as the proportion of variability in data explained by ANOVA. R^2^ values for the removal of HMF, furfural, and sugar were 0.9727, 0.9664, and 0.8755, respectively, with the relatively high values indicating that >87.5% of experimental data could be explained by the models (Table 4). 

**Figure 2:**
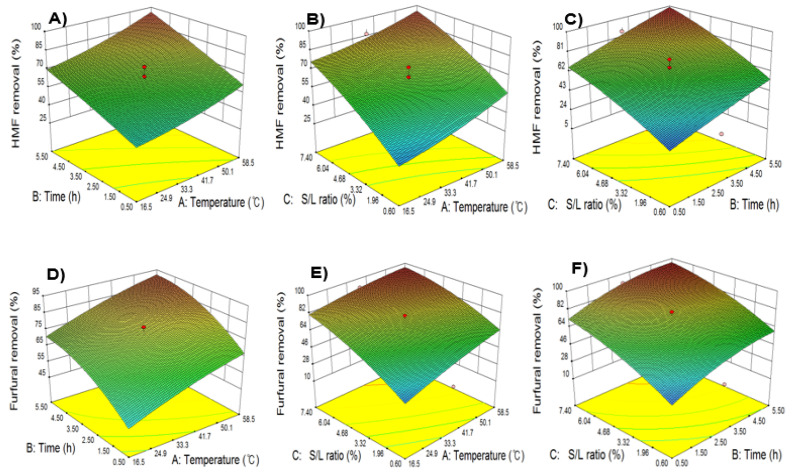
Response surface curves representing the effects of detoxification process conditions including extraction time, extraction temperature, and S-L ratio on 5-HMF and furfural removal. Time versus temperature (**A**,**D**); temperature versus S-L ratio (**B**,**E**); S-L ratio versus time (**C**,**F**).

To evaluate the interactive effect of independent variables on HMF and furfural removal rates using activated carbon, two variables were changed simultaneously and the other variable was fixed at the center point. When results were displayed as surface response curves, HMF and furfural removal rates tended to increase proportionally with all independent variables, indicating that 91%–96% of fermentation inhibitors could be removed. Removal rates of fermentation inhibitors tended to be similar, which indicated similar adsorption properties by inducing non-specific adsorption of 5-HMF and furfural by activated carbon. The S-L ratio had the greatest effect on HMF and furfural removal as shown in Figure 2. This was consistent with the results from the previous study, which showed that when using activated carbon, the removal of organic materials increases with treatment time and temperature [17]. Consistently, Ahmadi et al. reported that arsenic removal efficiency increases with an increase in temperature from 293 K to 323 K due to increased penetration and interaction in the outer boundary layer and pores of activated carbon, which is caused by the reduced viscosity of solvents and increased molecular movement [26]. In the detoxification experiment in APH using activated carbon, ≥96% fermentation inhibitors were removed by optimizing the detoxification variables, confirming that activated carbon could effectively remove fermentation inhibitors (Table 6). However, additional experiments regarding sugar adsorption by activated carbon were required, as the non-specific adsorption of sugars and fermentation inhibitors during detoxification could increase sugar loss and reduce ethanol yield [27].

### 2.5. Minimization of Sugar Loss

Experiments with acid catalyzed MAP showed that 1.17 g/L and 0.55 g/L of 5-HMP and furfural were produced during the pretreatment of *S. obliquus* (Table 1), respectively, and >96% of fermentation inhibitors were removed by detoxification using activated carbon, so the concentration of fermentation inhibitors has been reduced to less than 1 g/L, which is the level of microbial growth inhibition [28]. Despite the efficient removal of fermentation inhibitors, sugar removal occurred non-selectively, it is necessary to optimize detoxification conditions to minimize sugar loss and maximize removal of fermentation inhibitors simultaneously. 

The experimental results obtained from 17 experiments are shown in Table 6. The sugar removal rate ranges from 0.88% to 3.78%, which corresponds to 0.36 to 1.56 g/L of sugar in APH. By contrast, the maximum removal rates for 5-HMF and furfural were 97.4% and 96.1% in 17 experimental sets, indicating that 1.14 g/L and 0.53 g/L were removed by activated carbon, respectively (Table 1). These results indicate that limited amounts, up to 1.56 g/L, of sugar and fermentation inhibitors were adsorbed due to the adsorption saturation of activated carbon [29]. In terms of coded values, predicted responses for sugar removal were expressed using a second-order regression equation through multiple regression analysis (Table 3). For sugar removal, R^2^ was 0.876, which means that 87.6% of total variation on sugar removal data can be described by the developed models and *p*-values < 0.05 indicated that model terms were significant. According to the ANOVA results, experimental variables X_2_ and X_3_ had significant effects on sugar removal. 

**Figure 3:**
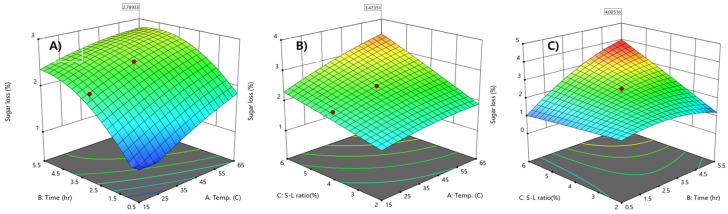
Response surface curves representing the effects of three variables, including time, temperature, and S-L ratio on sugar loss from APH of *S. obliquus*. Time versus temperature (**A**); temperature versus S-L ratio (**B**), time versus S-L ratio (**C**).

The response surface curve was developed for the evaluation of sugar removal from APH with varying extraction temperatures and times at a fixed S-L ratio of 4.0% (Figure 3A). Sugar removal increased with extraction temperatures and a maximum sugar removal of 2.78% was obtained with increased temperatures and times within the chosen experimental range. The response surface curve in Figure 3B shows sugar removal of APH as a function of extraction temperature and S-L ratio. Higher sugar removal was obtained with a higher extraction temperature and a higher S-L ratio.

An increase in the extraction temperature enhanced the sugar removal of APH due to the effects of temperature on mass transfer, such as an increase in the diffusion coefficient and the mobility of molecules between liquid phase and the adsorption surface. Adsorption is spontaneous and exothermic, and is expected to decrease with temperature increase. However, studies have also found the opposite or no relationship between changes in temperature and the amount of adsorbed substance [30]. Studies on the effect of temperature on 2,4-D adsorption using activated carbon have reported that surface activation and the breaking of existing bonds causes new active sites on the carbon surface, thereby increasing its adsorption efficiency. Furthermore, increasing the temperature improves the mobility of 2,4-D molecules from the liquid phase to the adsorption surface, increases penetration within the carbon surface and pores, and increases the rate of intermolecular diffusion [20,31].

### 2.6. Optimal Conditions for Detoxification

**Figure 4:**
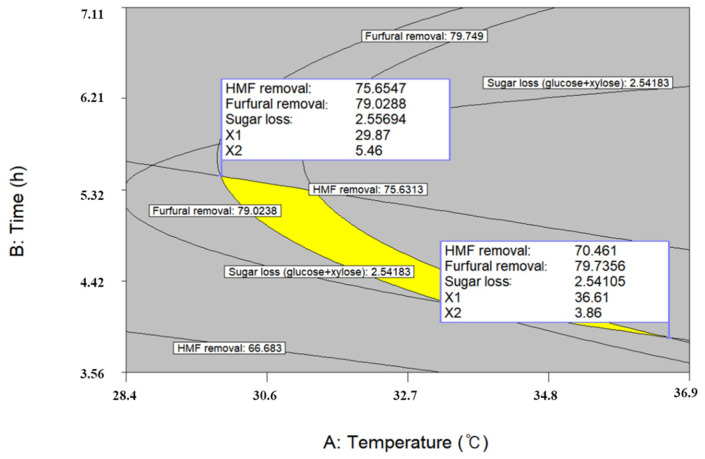
Superimposing contour map of optimized conditions for the maximum removal of HMF and furfural with the minimum sugar loss in APH of *S. obliquus*.

In the previous experiment, it was confirmed that the fermentation inhibitor could be effectively removed using activated carbon without significant sugar loss, and further optimization is required to minimize sugar loss and maximize fermentation inhibitor removal. Optimum conditions were obtained by superimposing of individual response surface curves, and the range of each detoxification condition was set at a temperature from 16.5 to 58.5 °C, a time from 0.5 to 5.5 h, and an S-L ratio from 0.6 to 7.4 %. As shown in Figure 4, the two optimal conditions were derived based on the criteria of minimizing extraction temperature and minimizing the extraction time, respectively. The latter condition was finally determined because short process time is more beneficial for reducing process costs. Then, the optimal detoxification conditions were predicted as 36.6 °C, 3.86 h, and 3.3%, respectively. Under these conditions, the removal of 70.5%, 79.7%, and 2.54% of 5-HMF, furfural, and sugar was predicted, respectively. To verify the predicted values obtained from the statistically-based optimization, a validation experiment was conducted. The removal rates of 5-HMF, furfural, and sugar were 71.6%, 83.1%, and 2.44%, respectively, showing similar results to the predicted values. Therefore, the prediction of the optimal value using the second-order regression models was valid for the optimization of a detoxification process using activated carbon.

### 2.7. Ethanol Fermentation

**Figure 5:**
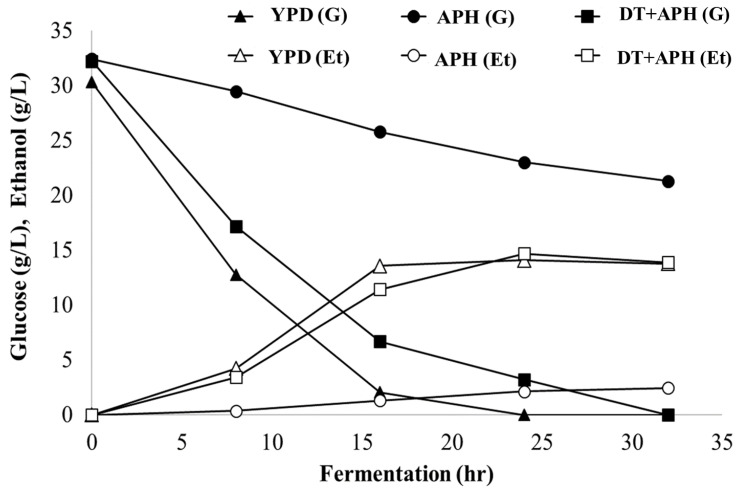
Fermentation using APH and detoxified APH (DT+APH) by *S. cerevisiae* for the production of ethanol. Glucose consumption of fermentation by *S. cerevisiae* in control with yeast-based medium—YPD (G, ▲), acid pretreated hydrolysate—APH (G, ●), detoxified acid pretreated hydrolysate—DT + APH (G, ■), ethanol production in YPD—YPD (Et, △), acid pretreated hydrolysate—APH (Et, ○), detoxified acid pretreated hydrolysate DT+APH (Et, □).

Detoxification of APH was conducted using activated carbon under optimum conditions of 36.6 °C, 3.86 h, and 3.3% prior to ethanol fermentation. In the fermentation experiment, a yeast strain, which uses glucose as the main carbon source for ethanol fermentation, was employed because the concentration of xylose in APH was relatively low and the need for xylose fermentation was not significant. To assess the effect of the major fermentation inhibitors present in APH on ethanol fermentation, control (YPD), acid pretreated hydrolysate (APH), and detoxified APH (DT-APH) were used as media to compare ethanol fermentation and sugar consumption by *Saccharomyces cerevisiae* KCTC 1126 (Figure 5). 

In ethanol fermentation using APH and YPD, the theoretical ethanol yields of 38.5% and 89.3% were obtained, respectively, which confirmed that the fermentation inhibitor in APH adversely affected the ethanol fermentation. After detoxification, acid hydrolysate (DT + APH) produced 13.9 g/L of ethanol, showing a theoretical yield of 84.5%, indicating that the produced ethanol concentration was comparable to the control group (YPD). The production of ethanol was 5.7 times higher than that obtained from APH, indicating that the removal of 5-HMF and furfural increased the production of ethanol. Lee et al. reported that, in the presence of 1–2 g/L of 5-HMF, the growth of *S. cerevisiae* is rapidly inhibited, and the theoretical yield of ethanol is between 15% and 35%, which is consistent with our results [14]. The glucose concentration profile during fermentation showed that fermentation inhibitors drastically inhibited glucose consumption during the 32 h of fermentation compared to YPD, consuming 11.1 g/L of glucose compared with 30.3 g/L, respectively. However, when APH was treated with activated carbon, glucose consumption improved and was comparable to that of YPD at 32 h. 

## 3. Materials and Methods

### 3.1. Microalgae Strain and Cultivation Conditions 

The green microalgae *S. obliquus* used in this study was provided by Library of Marine Samples (Geojesi, Gyeongnam, Korea). *S. obliquus* was cultivated in modified Bold medium (MBM) containing NaNO_3_ (0.25 g), CaCl_2_·2H_2_O (0.025 g), MgSO_4_·7H_2_O (0.075 g), K_2_HPO_4_ (0.075 g), KH_2_PO_4_ (0.175 g), NaCl (0.025 g), thiamine (1 mL, 6.5 mmol), and biotin (1 mL, 0.1 mmol) per liter of medium. The inoculum was cultured in a light–dark cycle of 12 h at 80 rpm in 250 mL Erlenmeyer flasks including 50 mL of MBM. The main culture was incubated at 25 °C, 60 μmol photons/m^2^ s^1^, and a light–dark cycle of 12 h in 1 L Erlenmeyer flasks with 200 mL of MBM.

### 3.2. Composition Determination

The compositional analysis of cellulose, hemicellulose, and lignin in *S. obliquus* was carried out according to the protocol TP-510-42618 published by NREL [32]. A 0.3 g of dried *S. obliquus* was hydrolyzed with 3 mL of 72% sulfuric acid at 30 °C for 1 h, and then subjected to a second hydrolysis with 87 mL of 4% sulfuric acid at 121 °C for 1 h. After two-step acid hydrolysis, the hydrolysate was filtered and analyzed for glucose and xylose using HPLC (Agilent, Santa Clara, CA, USA) [33]. The ash content was measured according to AACC method 08-01 [34]. A 2 g of dried sample was placed in a 550 °C muffler (WiseTherm, Daihan Scientific Co., Seoul, Korea) and incinerated for 8 h. After cooling, the weight of the sample was measured and the ash content was calculated. The lipid content of *S. obliquus* was determined using petroleum ether (Duksan, Ansan, Gyeonggi, Korea) at 70 °C in a Soxhlet extractor according to AACC approved method 30-25 [35]. A 10 g sample was used to extract lipids with 150 mL of petroleum ether, boiled in the temperature range of 35–60 °C for 16 h, and used at solvent condensation rates of 2–3 drops/sec.

### 3.3. Microwave-Assisted Pretreatment (MAP)

A 1 g of *S. obliquus* dry powder was placed in a tube and mixed with 10 mL of 0.2 M sulfuric acid (Merck, Kenilworth, USA) soaked for 10 min, and then placed in a microwave extractor (Multiwave 3000, Anton Paar, Graz, Austria). Electromagnetic waves were irradiated for 2 min at a power of 600 W. After extraction, the solid–liquid mixture was centrifuged at 5000 g for 10 min (LoboGene 1236R, Gimpo, Korea). The APH was then collected and filtered using a 0.45 µm membrane filter for the HPLC analysis of furfural, 5-HMF, and sugars. Samples were kept in a tightly closed bottle at below −4 °C for further analysis.

### 3.4. Experimental Design

A central composite design (CCD) was used to evaluate the interactive effects of independent variables for the maximization of removal of 5-HMF and furfural. As independent variables, the three variables chosen were extraction temperature (16.5–58.5 °C, X_1_), extraction time (0.5–5.5 min, X_2_), and S-L ratio (0.6–7.4 *w*/*v*%, X_3_). The Design-Expert software was used to establish a mathematical model and obtain the optimum conditions of the detoxification process. The independent variables were coded according to the following Equation (1):

(1) $$X_{i}=\frac{\left({ꭓ}_{i\;}-{ꭓ}_{0}\right)}{∆{ꭓ}_{i}}$$

where *X_i_* is the dimensionless value of an independent variable, *ꭓ_i_* is the real value of an independent variable, *ꭓ*_0_ is the real value of an independent variable at the center point, and Δ*ꭓ_i_* is the step change of the real value of the variable *i* corresponding to a variation of a unit for the dimensionless value of the variable *i*.

The experimental values of the 17 different runs were fitted to generate a second-order regression equation as following Equation (2):

*Y* = β_0_ + ∑β_i_*ꭓ*_i_ + ∑β_ij_*ꭓ*_i_*ꭓ*_j_ + ∑β_ii_*ꭓ*_i_^2^ (2)

where *Y* indicates the response, β_0_ represents the constant coefficient, β_i_ represents the slope or linear effect of the input variables *ꭓ*_i_, β_ij_ represents the interaction effect terms between the input variables *ꭓ*_i_ and *ꭓ*_j_, and β_ii_ represents the quadratic effect of input variable *ꭓ*_i_

Then, surface response plots were used for graphical analyses of the data to determine the interaction between the process variables and the responses. Design-Expert 8.0 software was used for the regression analysis of experimental data. The statistical significance was evaluated by the F-test, and the accuracy of the regression model was examined by the coefficient of determination (R^2^). The significant model terms were determined by the probability value (*p*-value) at a 95% confidence level [36].

### 3.5. Microorganism

The yeast strain, *Saccharomyces cerevisiae* KCTC 1126, was purchased from the Korean Biological Resources Center (Daejeon, Chungnam, Korea) and used for ethanol fermentation. The activation of yeast was carried out by adding dry yeast powder into warm distilled water in a 15 mL culture tube. Yeast cultures were maintained in a yeast-based medium (YPD) containing 1% yeast extract, 1% peptone, and 2% glucose at 25 °C for 24 h. The initial pH of the culture media was adjusted to 6.0 prior to sterilization at 121 °C for 15 min. Streak plating of the *S. cerevisiae* was carried out for two cycles to obtain a pure yeast culture, and the Petri dishes were placed in an incubator (JSGI-150T, JS Research Inc., Gongju, Korea) at 37 °C for 48 h before being further used in fermentation.

### 3.6. Fermentation

Yeast for inoculation was cultivated in 250 mL Erlenmeyer flasks filled with 50 mL of YPD medium containing 10 g/L yeast extract, 20 g/L peptone, 20 g/L glucose. After incubation at 30 °C and 200 rpm for 16 h, the yeast cells were inoculated at 1.0 × 10^7^ cells/mL into the culture medium to initiate the fermentation. The ethanol fermentation was performed in a 500 mL stirred fermenter (FM-500, Fermentech, Osong, Korea) with a 250 mL working volume. The batch fermentations were carried out in YPD medium at pH 6.5. The culture was agitated at 500 rpm for 32 h and the aeration rate was set at 1.5 vvm for aerobic growth of the yeast and adjusted to 1.0 vvm after 12 h to provide a more anaerobic environment for ethanol fermentation. The pH value was adjusted at 6.0–6.5 using 2 M NaOH. Cell densities (OD600) were determined using a spectrophotometer (Optizen 2120UV, Mecasys Co., Daejeon, Korea). Samples were harvested at 8 h intervals from the fermenter for the analysis of ethanol and glucose concentrations by GC and HPLC, respectively. 

### 3.7. Analysis Using GC and HPLC

The ethanol concentration was determined by gas chromatography (GC-14B, Shimadzu, Kyoto, Japan) using a polyethylene glycol (PEG-20 M) pack column with a flame ionization detector (FID). The glucose and xylose concentrations were measured by HPLC (Agilent 1200, Agilent Technol., Santa Clara, CA, USA) using an Aminex HPX-87H column (300 × 7.8 mm, Bio-Rad, Hercules, CA, USA) with a refractive index detector (RID-10A) at 50 °C. Sulfuric acid (5 mM) was used as a mobile phase at a flow rate of 0.5 mL/min. A Supelcosil LC-18 (SAS Institute Inc., Cary, NC, USA) stainless-steel column (250 × 4.6 mm) was used for the determination of 5-HMF and furfural. Analyses were carried out isocratically at room temperature with acetonitrile–water (50:50, *v/v*) as a mobile phase at a flow rate of 1 mL/min. All samples, with injection volume set at 20 μL, were filtered through a 0.22 μm filter and then injected into the HPLC. 

## 4. Conclusions

This study showed that fermentable sugar was produced through acid hydrolysis of *S. obliquus* and ethanol fermentation successfully carried out through the removal of fermentation inhibitors using activated carbon. The green microalga, *S. obliquus,* was an excellent candidate for bioethanol production due to its high starch and cellulose content and low lignin content compared to terrestrial plants. However, fermentation inhibitors such as 5-HMF and furfural were produced during pretreatment under acidic conditions, and the detoxification process was required to remove the fermentation inhibitors. Optimized detoxification conditions were predicted using statistically-based optimization, CCD, and process conditions were established to minimize sugar loss while maximizing the removal of fermentation inhibitors. When detoxification was performed under optimal conditions, the concentration of the fermentation inhibitors decreased from 1.72 g/L to 0.05 g/L, and the ethanol yield increased from 38.5% to 84.5%, confirming that detoxification by activated carbon was highly effective. Due to the higher carbohydrate content and higher pretreatment efficiency of *S. obliquus*, ethanol production was more economical as it enabled more efficient fermentation sugar production than 2^nd^ generation lignocellulosic biomass. 

## Figures and Tables

**Table 1:** The contents of the main components of *S.* *obliquus* and the concentrations of monomers in APH.

Components	S.obliquus * (g/100 g Dry Weight)	APH ** (g/L)	Conversion Rate *** (%)
Cellulose Starch	30.8 13.8	38.1 (glucose)	77.8
Hemicellulose	4.9	3.3 (xylose)	61.7
Lignin	1.9	-	-
Protein Ash	15.5 14.2	- -	- -
Lipid 5-HMF Furfural	9.7 - -	- 1.17 0.55	- 2.51 6.94

* composition of dried *S.* *obliquus* before acid pretreatment. ** conversion rates to monomeric sugar (glucose and xylose) and toxic byproduct (5-HMF and furfural) based on concentrations in APH*; Solid-liquid ratio of pretreatment = 1:10. *** $\mathrm{Glucose}\;\mathrm{conversion}\;\mathrm{rate}\;\left(\%\right)=\frac{Glucose\;conc.\;in\;hydrolysate\left(\frac{g}{L}\right)}{\mathrm{Cellulose}\;\mathrm{content}\;\left(\frac{g}{L}\right)\;\times \;1.1}\;\times \;100$, *** $\;\mathrm{Xylose}\;\mathrm{conversion}\;\mathrm{rate}\;\left(\%\right)=\frac{Xylose\;conc.\;in\;hydrolysate\;\left(\frac{g}{L}\right)}{\mathrm{Hemicellulose}\;\mathrm{content}\;\left(\frac{g}{L}\right)\;\times \;1.14}\;\times \;100$

**Table 2:** Composition of cellulose, hemicellulose, and lignin in terrestrial lignocellulosic feedstock [18].

	Dry Weight (%)		
	Cellulose	Hemicellulose	Lignin
**Corn stover**	37.5	22.4	17.6
**Corn cob**	45.0	35.0	12.0
**Corn fiber**	14.3	16.8	8.4
**Barley straw**	41.6	19.6	17.7
**Rice straw**	35.0	25.0	12.0
**Wheat straw**	38.2	21.2	23.4
**Sugarcane bagasse**	40.0	24.0	25.0
**Switchgrass**	31.0	20.4	17.6
**Pine tree**	46.4	8.8	29.4
**Poplar**	49.9	17.4	18.1

**Table 3:** Experimental ranges and coded levels of independent variables for central composite design.

Variables		Coded and Actual Levels				
		−1.68	−1	0	+1	+1.68
X_1_	Temperature (°C)	16.5	25.0	37.5	50	58.5
X_2_	Time (h)	0.5	1.5	3.0	4.5	5.5
X_3_	^1^ S-L ratio (*w/v*%)	0.6	2.0	4.0	6.0	7.4

^1^ Solid–liquid ratio (%) = the percent weight ratio of activated carbon added to APH.

**Table 4:** Second-order polynomial equations calculated by central composite design for the optimization of the detoxification process.

Responses	Second-Order Polynomials	R^2^	P
5-HMF removal	Y_1_ = 70.180 + 6.43X_1_ + 8.15X_2_ + 20.792X_3_ + 0.2697X_1_X_2_ − 1.25X_1_X_3_ + 0.1547X_2_X_3_ − 0.7983X_1_^2^ − 1.40X_2_^2^ − 5.25X_3_^2^	0.9727	0.0001
Furfural removal	Y_2_ = 76.58 + 6.34X_1_ + 7.46X_2_ + 18.43X_3_ − 0.0276X_1_X_2_ − 1.97X_1_X_3_ − 0.4870X_2_X_3_ − 0.6308X_1_^2^ − 2.11X_2_^2^ − 5.18X_3_^2^	0.9664	0.0021
Sugar loss	Y_2_ = 2.38 + 0.1962X_1_ + 0.3891X_2_ + 0.5595X_3_ − 0.0488X_1_X_2_ + 0.1037X_1_X_3_ + 0.5012X_2_X_3_ − 0.0204X_1_^2^ − 0.1406X_2_^2^ − 0.0187X_3_^2^	0.8755	0.0178

**Table 5:** Analysis of variance of the experimental results of central composite design for second-order polynomial models.

	HMF Removal (%)			Furfural Removal (%)			Sugar Loss (%)		
	Sum of Squares	*F* Value	*p* Value	Sum of Squares	*F* Value	*p* Value	Sum of Squares	*F* Value	*p* Value
Model	7708.43	27.76	0.0001	6302.57	22.35	0.0002	9.22	5.47	0.0178
X_1_	564.70	18.30	0.0037	549.22	17.53	0.0041	0.5259	2.81	0.1377
X_2_	907.51	29.41	0.0010	759.15	24.23	0.0017	2.07	11.04	0.0127
X_3_	5905.02	191.39	<0.0001	4640.72	148.10	<0.0001	4.27	22.82	0.0020
X_1_X_2_	0.5819	0.0189	0.8946	0.0061	0.0002	0.9893	0.0190	0.1015	0.7593
X_1_X_3_	12.49	0.4047	0.5449	31.15	0.9941	0.3519	0.0861	0.4597	0.5195
X_2_X_3_	0.1915	0.0062	0.9394	1.90	0.0606	0.8127	2.01	10.73	0.0136
X_1_^2^	7.18	0.2328	0.6442	4.49	0.1431	0.7164	0.0047	0.0251	0.8785
X_2_^2^	22.23	0.7204	0.4241	50.37	1.61	0.2454	0.2230	1.19	0.3114
X_3_^2^	310.94	10.08	0.0156	302.34	9.65	0.0172	0.0039	0.0210	0.8889

X_1_: extraction temperature (°C); X_2_: extraction time (h); X_3_: S-L ratio (*w/v*%).

**Table 6:** Experimental design for the detoxification process and experimental data obtained from central composite design.

Run No.	X_1_	X_2_	X_3_	HMF Removal	Furfural Removal	Sugar Loss(Glucose + Xylose)
				(%)		
1	25.0	1.5	2.0	31.9	40.0	1.82
2	50.0	1.5	2.0	42.2	53.3	2.42
3	25.0	4.5	2.0	39.0	48.5	1.51
4	50.0	4.5	2.0	56.8	66.5	1.65
5	25.0	1.5	6.0	73.8	79.4	1.53
6	50.0	1.5	6.0	85.5	89.7	2.28
7	25.0	4.5	6.0	88.0	90.7	2.96
8	50.0	4.5	6.0	97.4	96.1	3.78
9	16.5	3.0	4.0	53.7	60.4	2.14
10	58.5	3.0	4.0	78.5	83.9	2.36
11	37.5	0.5	4.0	39.6	49.4	0.88
12	37.5	5.5	4.0	85.9	86.5	2.94
13	37.5	3.0	0.6	20.1	28.3	0.92
14	37.5	3.0	7.4	86.9	90.3	3.59
15	37.5	3.0	4.0	66.1	75.0	2.56
16	37.5	3.0	4.00	74.0	79.6	2.24
17	37.5	3.0	4.00	57.9	76.1	2.35

X_1_: extraction temperature (°C); X_2_: extraction time (h); X_3_: S-L ratio (*w*/*v*%).

## Data Availability

No new data were created or analyzed in this study. Data sharing is not applicable to this article.

## Sample Availability

Samples of the compounds are not available from the authors.
